# ﻿*Alseodaphnopsismaguanensis* is conspecific with *A.hokouensis* (Lauraceae) based on morphological and molecular evidence

**DOI:** 10.3897/phytokeys.242.115679

**Published:** 2024-06-03

**Authors:** Lang Li, Dian-Yang Zou, Ya-Meng Mao, Jie Li

**Affiliations:** 1 Plant Phylogenetics and Conservation Group, Center for Integrative Conservation & Yunnan Key Laboratory for Conservation of Tropical Rainforests and Asian Elephants, Xishuangbanna Tropical Botanical Garden, Chinese Academy of Sciences, Mengla, Yunnan 666303, China Xishuangbanna Tropical Botanical Garden, Chinese Academy of Sciences Yunnan China; 2 University of Chinese Academy of Sciences, Beijing 100049, China University of Chinese Academy of Sciences Beijing China

**Keywords:** *Alseodaphnopsis*, Lauraceae, morphology, phylogeny, synonym, taxonomy

## Abstract

Based on both morphological and molecular evidence, it is confirmed that *Alseodaphnopsismaguanensis* is conspecific with *A.hokouensis*. Hence, *Alseodaphnopsismaguanensis* is treated as a synonym of *A.hokouensis* here. The conservation status of *Alseodaphnopsishokouensis* is also re-evaluated according to the IUCN Red List Categories and Criteria in this study.

## ﻿Introduction

As one of the largest basal angiosperm families, Lauraceae includes more than 50 genera and 2500 ~ 3000 species distributed in tropical and subtropical regions worldwide (Chanderbali et al. 2001). The delimitation, delineation and identification of Lauraceae species, especially for tropical species, are always complicated due to limited variation in floral and other morphological characters and insufficient specimen collection (van der Werff and Richter 1996; Rohwer 2000; Li and Li 2004; Yang and Liu 2015).

*Alseodaphnopsishokouensis* (H. W. Li) H. W. Li & J. Li was first published as an *Alseodaphne* Nees species (Lee et al. 1979) and then it was transferred to *Alseodaphnopsis* H. W. Li & J. Li, a recently described genus of the Lauraceae (Mo et al. 2017). Since it was first collected in 1953, only three herbarium records (including two type specimens) of *Alseodaphnopsishokouensis* can be found and no fruiting specimen has been collected.

During recent field surveys in SE Yunnan (SW China), some flowering and fruiting individuals of *Alseodaphnopsishokouensis* were found and they resembled a recently-published *Alseodaphnopsis* species, *A.maguanensis* L. Li & J. Li (Li et al. 2020). Based on both morphological and molecular evidence, we confirm that *Alseodaphnopsismaguanensis* is conspecific with *A.hokouensis*. Therefore, we treat *Alseodaphnopsismaguanensis* as a synonym of *A.hokouensis* here. Based on the field survey data, we also re-evaluate the conservation status of *Alseodaphnopsishokouensis* in this study.

## ﻿Materials and methods

### ﻿Field surveys

We conducted field surveys in both Hekou (Hokou) County and Maguan County (Yunnan Province, China) from 2020 to 2022. Flowering specimens of *Alseodaphnopsishokouensis* and *A.maguanensis* were collected from May to June and fruiting specimens were collected from July to September.

### ﻿Morphological studies

Morphological characters of *Alseodaphnopsishokouensis* and *A.maguanensis* were examined and compared in detail, based on fresh and preserved materials as well as dried specimens collected in the field. Herbarium specimens of these two species from the Herbaria HITBC, KUN and PE were also examined.

### ﻿Molecular studies and phylogenetic analyses

Based on the work of Li et al. (2020), three individuals of *Alseodaphnopsishokouensis* and two additional individuals of *A.maguanensis* were sampled in the present study. DNA extraction, fragment amplification and sequencing, as well as DNA sequence alignment, followed the work of Li et al. (2020). The newly-obtained DNA sequences were integrated into the ITS + *LEAFY* intron II combined dataset of the work by Li et al. (2020). Species examined in this study, voucher information, collection locality and GenBank accessions for ITS and *LEAFY* intron II sequences are given in Table 1.

**Table 1.** Species examined in this study, voucher information, collection localities and GenBank accession numbers for ITS and *LEAFY* sequences.

Taxon	Voucher	Locality	ITS	*LEAFY*
**Ingroups**				
***Alseodaphne* (4)**				
*A.gigaphylla* Kosterm.	Arifiani DA657 (BO)	Indonesia, Java	HQ697181	HQ697004
*A.gracilis* Kosterm.	Li L. 20070187 (HITBC)	China, Yunnan	HQ697187	HQ697036
*A.huanglianshanensis* H. W. Li & Y. M. Shui	Li L. 20080006 (HITBC)	China, Yunnan	HQ6971812	HQ697007
*A.semecarpifolia* Nees	Arifiani DA658 (BO)	Indonesia, Java	HQ6971814	HQ697015
***Alseodaphnopsis* (8)**				
*A.andersonii* (King ex Hook. f.) H. W. Li & J. Li,	Li J. & Li L. 20070074 (HITBC)	China, Yunnan	FM957793	HQ697002
*A.hainanensis* (Merr.) H. W. Li & J. Li	Li L. & Wang Z. H. JFL07 (HITBC)	China, Hainan	MG188587	MG188634
	Li L. & Wang Z. H. LMS10 (HITBC)	China, Hainan	MG188586	MG188633
*A.hokouensis* (H. W. Li) H. W. Li & J. Li	Li L. et al. 2020064 (HITBC)	China, Yunnan	PP736795	PP737831
	Li L. et al. 2020071 (HITBC)	China, Yunnan	PP736796	PP737832
	Li L. et al. 2020072 (HITBC)	China, Yunnan	PP736797	PP737833
*A.maguanensis* L. Li & J. Li	Li L. et al. GLQ45 (HITBC)	China, Yunnan	MN906900	MN906896
	Li L. et al. GLQ46 (HITBC)	China, Yunnan	MN906901	MN906897
	Li L. et al. 2020086 (HITBC)	China, Yunnan	PP736798	PP737834
	Li L. et al. 2020091 (HITBC)	China, Yunnan	PP7367999	PP737835
*A.petiolaris* (Meisn.) H. W. Li & J. Li	Chen J. Q. 07003 (HITBC)	China, Yunnan	FM957796	HQ697008
*A.putaoensis* L. Li, Y. H. Tan & J. Li	Li L. & Ma H. MM254 (HITBC)	Myanmar, Kachin	MN906902	MN906898
	Li L. & Ma H. MM266 (HITBC)	Myanmar, Kachin	MN906903	MN906899
*A.rugosa* (Merr. & Chun) H. W. Li & J. Li,	Li L. & Wang Z. H. MYH02 (HITBC)	China, Hainan	MG188585	MG188635
	Li L. & Wang Z. H. MYH08 (HITBC)	China, Hainan	MG188584	MG188640
*A.sichourensis* (H. W. Li) H. W. Li & J. Li	Song Y. 33225 (HITBC)	China, Yunnan	MG188597	MG188626
*A.ximengensis* H.W. Li & J. Li	Li J. W. 1235 (HITBC)	China, Yunnan	MG188591	MG188599
***Dehaasia* (1)**				
*D.hainanensis* Kosterm.	Li L. & Wang Z. H. 20070373 (HITBC)	China, Hainan	FJ719308	HQ697026
***Machilus* (8)**				
*M.duthiei* King ex Hook. f.	Zhong J. S. 2006094 (HITBC)	China, Yunnan	FJ755425	HQ697055
*M.gongshanensis* H. W. Li	Chen J. Q. 07002 (HITBC)	China, Yunnan	FJ755416	HQ697047
*M.grijsii* Hance	Chen J. Q. et al. 2006028 (HITBC)	China, Guangdong	FJ755420	HQ697049
*M.kwangtungensis* Yang	Chen J. Q. et al. 2006027 (HITBC)	China, Guangdong	FJ755424	HQ697051
*M.monticola* S. Lee	Li L. & Wang Z. H. 20070323 (HITBC)	China, Hainan	FJ755418	HQ697057
*M.platycarpa* Chun	Chen J. Q. et al. 2006073 (HITBC)	China, Guangdong	FJ755421	HQ697067
*M.robusta* W. W. Sm.	Li J. 2002116 (HITBC)	China, Guangxi	FJ755426	HQ697071
*M.yunnanensis* Lec.	Zhong J. S. 2006093 (HITBC)	China, Yunnan	FJ755415	HQ697084
***Nothaphoebe* (1)**				
*N.umbelliflora* (Blume) Blume	Arifiani DA495 (BO)	Indonesia, Java	HQ697191	HQ697088
***Phoebe* (6)**				
*P.chekiangensis* C. B. Shang	Li J. & Li L. 20070188 (HITBC)	China, Zhejiang	FJ755407	HQ697128
*P.cuneata* (Blume) Blume	Arifiani 40 (MO)	Indonesia, Java	HQ697202	HQ697130
*P.formosana* (Hayata) Hayata	Rohwer 156 (MJG)	Germany, Bonn	HQ697205	HQ697136
*P.lanceolata* (Wall. ex Nees) Nees	Chen J. Q. et al. 2006093 (HITBC)	China, Guangdong	FJ755410	HQ697141
*P.glaucifolia* S. K. Lee & F. N. Wei	Chen J. Q. et al. 2005002 (HITBC)	China, Yunnan	FJ755409	HQ697150
*P.neurantha* (Hemsl.) Gamble	Li J. & Li L. 20070214 (HITBC)	China, Zhejiang	HQ697209	HQ697151
**Outgroups**				
***Actinodaphne* (1)**				
*A.trichocarpa* C. K. Allen	Li L. 20070282 (HITBC)	China, Sichuan	HQ697214	HQ697166
***Lindera* (1)**				
*L.erythrocarpa* Makino	Li J. & Li L. 20070203 (HITBC)	China, Zhejiang	HQ697215	HQ697167
***Litsea* (1)**				
*L.auriculata* Chien & Cheng	Li J. & Li L. 20070195 (HITBC)	China, Zhejiang	HQ697217	HQ697174
***Neolitsea* (1)**				
*N.howii* C. K. Allen	Li L. & Wang Z. H. 20070379 (HITBC)	China, Hainan	HQ697220	HQ697178

The combined dataset including ITS and *LEAFY* intron II sequences was used for phylogenetic analysis according to the works of Li et al. (2011), Mo et al. (2017) and Li et al. (2020). Phylogenetic analyses were performed using the Maximum Parsimony (MP) and Bayesian Inference (BI) methods. The MP analysis was performed using PAUP* 4.0b10 (Swofford 2003). The BI analysis was performed using MrBayes v.3.2.6 (Ronquist and Huelsenbeck 2003). Different DNA sequences were defined as separate data partitions. The evolutionary model for each partition (ITS: GTR+I+G; *LEAFY* intron II: HKY+G) was estimated using jModelTest v.2.1.10 (Darriba et al. 2012) with the Akaike Information Criterion (AIC) (Akaike 1974; Posada and Buckley 2004). The parameters used in both MP and BI analysis followed the work of Li et al. (2020).

## ﻿Results and discussion

**Figure 1.**
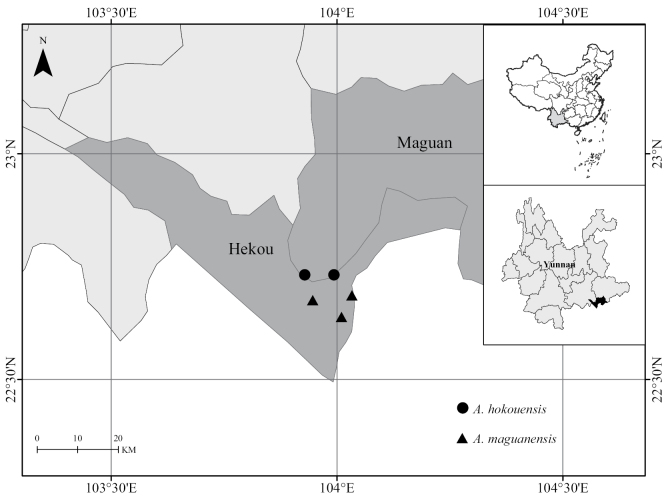
The recorded localities of *Alseodaphnopsishokouensis* and *A.maguanensis* in SE Yunnan, SW China.

During our field surveys in SE Yunnan, two populations of *Alseodaphnopsishokouensis* were found in Huayudong and Qincaitang, Nanxi Town, Hekou County (Fig. 1), each with about 10 mature individuals. No individual was found at the type locality of *Alseodaphnopsishokouensis*, Masike, Nanxi Town, Hekou County. Two populations of *Alseodaphnopsismaguanensis*, each with about 20 mature individuals, were also investigated in Gulinqing Provincial Natural Reserve (Maguan County) according to the work of Li et al. (2020).

**Figure 2.**
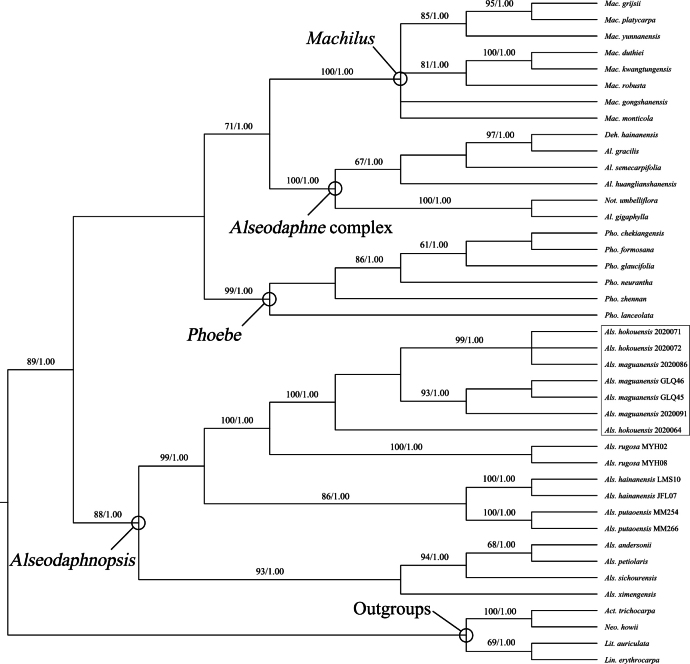
Bayesian consensus tree of ITS + *LEAFY* intron II combined dataset. MP bootstrap (BS ≥ 50%) and Bayesian posterior probability (PP ≥ 0.95) values are shown above branches. *Act.* = *Actinodaphne*, *Al.* = *Alseodaphne*, *Als.*= *Alseodaphnopsis*, *Deh.* = *Dehaasia*, *Lin.* = *Lindera*, *Lit.* = *Litsea*, *Mac.* = *Machilus*, *Neo.* = *Neolitsea*, *Not.* = *Nothaphoebe*, *Pho.* = *Phoebe*.

The MP and BI analyses of the ITS + *LEAFY* intron II combined dataset generated congruent topologies. The Bayesian consensus tree with MP bootstrap (BS) and Bayesian posterior probability (PP) values is shown in Fig. 2. As in the work of Li et al. (2020), all *Alseodaphnopsis* specimens sampled in the present study formed a well-defined clade (BS 88%, PP 1.00). Within the *Alseodaphnopsis* clade, there are two well-supported subclades which consist of four and five species, respectively. All individuals of *Alseodaphnopsishokouensis* and *A.maguanensis* formed a strongly-supported clade (BS 100%, PP 1.00), closely related to *A.rugosa*. Within this clade, the individuals of these two species are mixed with each other and their relationships are poorly resolved.

**Figure 3.**
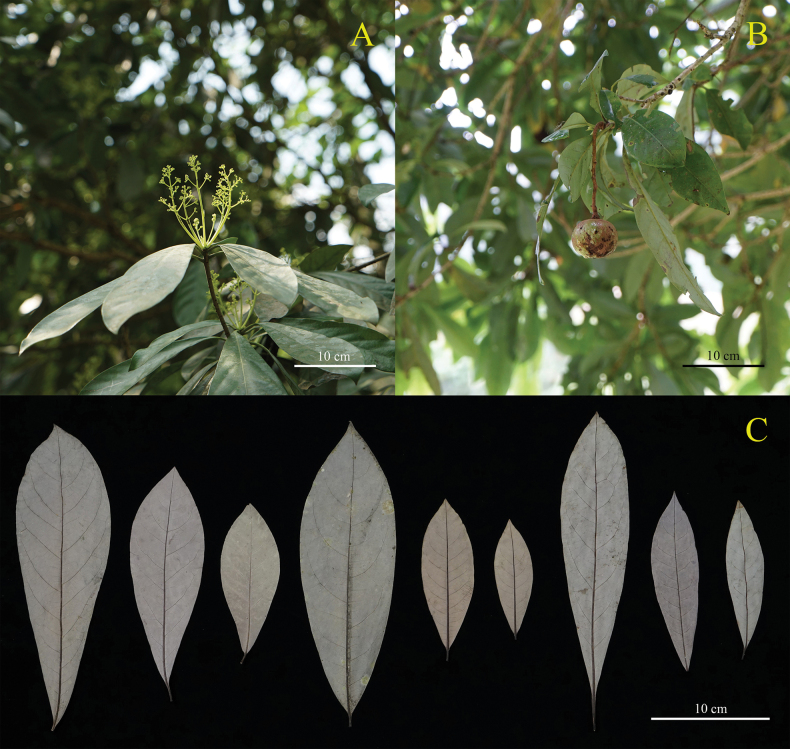
*Alseodaphnopsishokouensis***A** flowering branchlet **B** fruiting branchlet **C** different shapes of leaf blades. Photographed by Lang Li.

The morphological characters of *Alseodaphnopsishokouensis*, *A.maguanensis* and closely related *A.rugosa* were compared in detail, based on the data from type specimens and protologues (Li et al. 2008; Li et al. 2020). Morphological comparisons showed that *Alseodaphnopsismaguanensis* is almost the same as *A.hokouensis*, except that the leaves of the former are oblong-obovate or oblong-oblanceolate and those of the latter are elliptic to oblong (Table 2). After carefully checking the specimens collected from field surveys, we found that the leaves of both species can vary from elliptic, oblong to oblong-obovate or oblong-oblanceolate (Fig. 3). They are usually oblong-obovate or oblong-oblanceolate and occasionally elliptic to oblong. According to the newly-obtained fruiting specimens of *Alseodaphnopsishokouensis*, the fruit characters of *Alseodaphnopsishokouensis* are also consistent with those of *A.maguanensis* (Fig. 3). Additionally, the phenologies and habitats of these two species are quite similar and their distribution areas are adjacent as well (Fig. 1).

**Table 2.** Comparison of morphological characters, phenologies, habitats and distributions of *Alseodaphnopsishokouensis*, *A.maguanensis* and *A.rugosa*.

	*A.hokouensis*	*A.maguanensis*	*A.rugosa*
**Morphological characters**			
Branchlet	terete, brownish when dry, striate, glabrous	terete, greyish, wrinkled, glabrous, with lenticels and leaf scars	terete, robust, wrinkled, with dense leaf scars near top
Terminal bud	subglobose, bud scales broadly ovate, acute at apex, glabrous	subglobose, bud scales broadly ovate, acute at apex, glabrous	
Leaf arrangement	leaves alternate	leaves clustered at apex of branchlet, alternate or subverticillate	leaves inserted at apex of branchlet, dense and nearly verticillate
Petiole	1.5–3 cm, concave-convex, glabrous	1.5–2.5 cm, concave-convex, glabrous	robust, 1.5–2.5 cm, glabrous
Leaf blade	elliptic to oblong, 10.5–17 × 4–6.5 cm, subleathery, glabrous on both surfaces, base broadly cuneate to subrounded, apex abruptly shortly acuminate	oblong-obovate or oblong-oblanceolate, 12–32 × 3.5–9 cm, leathery, glabrous on both surfaces, base cuneate, apex shortly acuminate	oblong-obovate or oblong-oblanceolate, 15–36 × 4–10 cm, leathery, glabrous on both surfaces, base cuneate, apex shortly acuminate.
Leaf veins	mid-rib elevated abaxially, impressed adaxially, lateral veins 9–13 pairs, transverse veins and veinlets densely reticulate, conspicuous on both surfaces when dry	mid-rib elevated abaxially, impressed adaxially, lateral veins 8–12 pairs, veins and veinlets conspicuous, reticulate, elevated on both surfaces when dry	mid-rib conspicuously elevated abaxially, impressed adaxially, veins and veinlets conspicuous, reticulate
Inflorescence	panicles subterminal or inserted on lower part of young branchlet, 10.5–15 cm, peduncle and rachis glabrous, pedicels 3–4 mm, glabrous	panicles subterminal, 15–20 cm, peduncle glabrous, pedicels 5–8 mm, glabrous	not seen
Perianth lobes	perianth lobes 6, ovate, slightly acute, subequal, ca. 2 × 1.5 mm, glabrous outside, grey pubescent inside, deciduous	perianth lobes 6, broadly ovate, acute, subequal, outer ones ca. 2 × 1.5 mm, inner ones ca. 2.5 × 2 mm, glabrous outside, white pubescent inside, deciduous	not seen
Fertile stamens	fertile stamens 9, minute, ca. 1.5 mm in 1^st^ and 2^nd^ whorls, ca. 1.7 mm in 3^rd^ whorl; filaments villous, ca. 0.7 mm in 1^st^ and 2^nd^ whorls, ca. 1 mm in 3^rd^ whorl, those of 3^rd^ whorl each with 2 stalked orbicular-reniform glands at base, others glandless; anthers of 1^st^ and 2^nd^ whorls oblong, almost as long as filament, with introrse cells, those of 3^rd^ whorl rectangular, with extrorse cells	fertile stamens 9, ca. 2 mm in 1^st^ and 2^nd^ whorls, ca. 2.2 mm in 3^rd^ whorl; filaments villous, those of 3^rd^ whorl each with 2 shortly-stalked orbicular-cordate glands at base, others glandless; anthers of 1^st^ and 2^nd^ whorls ovate, almost as long as filament, cells all introrse, those of 3^rd^ whorl elliptic, with extrorse cells	not seen
Staminodes	not seen	ca. 1.5 mm, sagittate, stalked	not seen
Pistil	ovary ovoid, ca. 1.5 mm, glabrous, attenuate into a ca. 0.5 mm long style; stigma discoid, slightly lobed	ovary ovoid, ca. 1.2 mm, glabrous, attenuate into a ca. 0.8 mm long style; stigma discoid, inconspicuous	not seen
Infructescence	not seen	subterminal, 10–18 cm, robust, glabrous, with only one or two well-developed fruits	subterminal, ca. 12.5 cm, robust, glabrous
Fruit	not seen	oblate, 4–5 × 5–6 cm, brown when mature; fruit stalk robust, 3–4 mm in diameter, apex dilated, 5–10 mm in diameter, sometimes nearly cylindrical, fleshy and warty when fresh	oblate, ca. 2.5 × 3 cm deep purple or black when mature; fruit stalk robust, 5–8 mm in diameter on top, fleshy, red and warty when fresh
**Phenology**	flowering in May; fruit unknown	flowering in May–Jun; fruiting in Jul–Sep	flower unknown; fruiting in Jul–Dec (fruits mostly found in Oct–Dec)
**Habita**t	evergreen broad-leaved forests; ca. 700 m alt.	tropical montane forests in valleys; ca. 800 m alt.	mixed forests in valleys; 1200–1300 m alt.
**Distribution**	SE Yunnan, China	SE Yunnan, China	Hainan, China

To sum up, the results of both morphological comparison and phylogenetic analysis showed that *Alseodaphnopsismaguanensis* is conspecific with *A.hokouensis*. Therefore, we propose that *Alseodaphnopsismaguanensis* should be treated as a synonym of *A.hokouensis*. The insufficient specimen collection of *Alseodaphnopsishokouensis*, especially the lack of fruiting specimens, hinders a comprehensive understanding of the species. The same situation is found in many other rare and endemic Lauraceae species in SE Yunnan, such as species of *Alseodaphne*, *Beilschmiedia* Nees, *Caryodaphnopsis* Airy Shaw, *Cryptocarya* R. Br., *Endiandra* R. Br., *Machilus* Rumph. ex Nees, *Phoebe* Nees and *Syndiclis* Hook. f., etc. (Li et al. 2008). Thus, more field surveys and specimen collections are suggested in SE Yunnan in order to improve the understanding of these rare and endemic Lauraceae species.

### ﻿Taxonomic treatment

#### ﻿ Alseodaphnopsis hokouensis

Taxon classificationPlantaeLauralesLauraceae

(H. W. Li) H. W. Li & J. Li

5AAF0A7D-40FF-5373-A780-3E21F937DEA8

Alseodaphne hokouensis H. W. Li, Act Phytotax. Sin. 17 (2): 71. 1979. Basionym. Type: CHINA. Yunnan Province: Hekou County, 10 May 1953, K. H. Tsai *1039* (holotype: KUN [KUN0047456!]; isotype: KUN [KUN0108581!]).
= Alseodaphnopsismaguanensis L. Li & J. Li, PhytoKeys 138: 27–39. 2020. **syn. nov.** Type: CHINA. Yunnan Province: Maguan County, 14 May 2016, Lang Li et al. *GLQ26* (holotype: HITBC!).

##### Revised description.

Trees evergreen, up to 20 m tall. Branchlets terete, 3–6 mm in diameter, greyish, striate, glabrous, with lenticels and leaf scars. Terminal buds subglobose, ca. 2 mm in diameter; bud scales broadly ovate, acute at apex, glabrous. Leaves clustered at apex of branchlet, alternate or subverticillate; petiole robust, 2–3 mm thick, 1.5–3 cm long, concave-convex; leaf blade green adaxially, glaucous abaxially when young, but green or pale green when mature, oblong-obovate or oblong-oblanceolate, sometimes elliptic to oblong, 10.5–32 × 3.5–9 cm, subleathery to leathery, glabrous on both surfaces, mid-rib elevated abaxially, impressed adaxially, lateral veins 8–13 pairs, slightly elevated on both surfaces, oblique, evanescent and interconnected near leaf margin, transverse veins and veinlets densely reticulate, conspicuous on both surfaces when dry, base cuneate to broadly cuneate, apex shortly acuminate, sometimes abruptly shortly acuminate. Panicles subterminal, clustered at apex of branchlet, 10.5–20 cm, many-flowered; peduncle branched at middle or above, peduncle and rachis glabrous; bracts and bracteoles linear, ca. 1.5 mm, acute, ciliate, caducous. Pedicels slender, 3–8 mm, slightly dilated on top, glabrous. Flowers small, ca. 2.5 mm. Perianth tube short; perianth lobes 6, broadly ovate, slightly acute, glabrous outside, pubescent inside, subequal, outer ones ca. 2 × 1.5 mm, inner ones ca. 2.5 × 2 mm, deciduous. Fertile stamens 9, minute, 1.5–2 mm in 1^st^ and 2^nd^ whorls, 1.7–2.2 mm in 3^rd^ whorl; filaments villous, 0.7–1 mm in 1^st^ and 2^nd^ whorls, 1–1.2 mm in 3^rd^ whorl, those of 3^rd^ whorl each with 2 shortly stalked orbicular-cordate glands at base, others glandless; anthers of 1^st^ and 2^nd^ whorls ovate, almost as long as filaments, with introrse cells, those of 3^rd^ whorl elliptic, slightly shorter than filaments, with extrorse cells. Staminodes conspicuous, ca. 1.5 mm, sagittate, stalked. Ovary ovoid, 1.2–1.5 mm, glabrous, attenuate into a 0.5–0.8 mm long style; stigma discoid, inconspicuous. Infructescence subterminal, 10–18 cm, robust, glabrous, with one or two well-developed fruits. Fruit oblate, 4–5 × 5–6 cm, immature fruit green, brown when mature, fruit stalk robust, 3–4 mm in diameter, apex dilated, 5–10 mm in diameter, sometimes nearly cylindrical, fleshy and warty when fresh.

##### Phenology.

Flowering from May to June and fruiting from July to September.

##### Distribution and habitat.

Hekou County and Maguan County, Yunnan Province, China. Tropical limestone forests in valleys, usually near streams, at an elevation of 150–850 m.

##### Conservation status.

**Figure 4.**
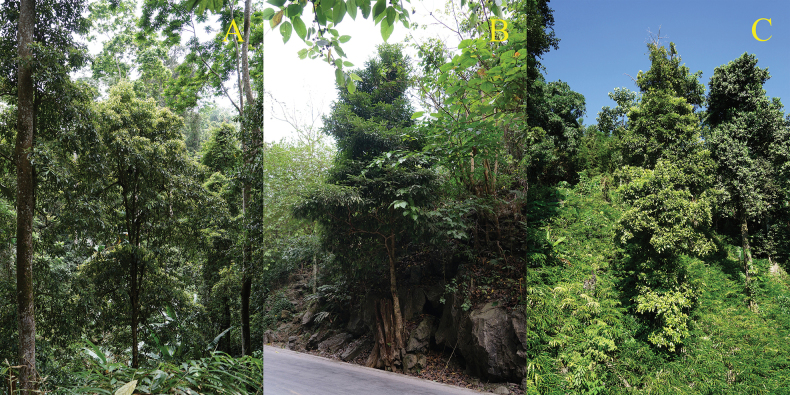
Different habitats of *Alseodaphnopsishokouensis***A** in the forest of the Nature Reserve **B** on the roadside **C** in the strongly disturbed forest near the village.

Currently, *Alseodaphnopsishokouensis* is known from Hekou Country and Maguan Country (Yunnan Province, China) with four populations (Fig. 1). Two populations found in Maguan Country are all located in Gulinqing Provincial Nature Reserve, each with about 20 mature individuals (Fig. 4). The other two populations found in Hekou Country, each with about 10 mature individuals, are not located in any nature reserve and those individuals mostly occur on the roadsides or in strongly-disturbed forests near the villages (Fig. 4). No individual was found at the type locality, Masike, Nanxi Town, Hekou County. According to the IUCN Red List Categories and Criteria version 15.1 (July 2022), the conservation status of *Alseodaphnopsishokouensis* is re-evaluated as Critically Endangered (CR, C2a(i)).

##### Additional specimens examined.

China. Yunnan Province: **Hekou County**, 17 June 2020, Lang Li et al. *2020064* (HITBC); 18 June 2020, Lang Li et al. *2020071* & *2020072* (HITBC); 25 August 2020, Lang Li and Guan-long Cao, *2020135*, *2020136*, 2020137 & *2020138* (HITBC). 13 May 2022, Lang Li and Dian-yang Zou, *2022027* (HITBC). **Maguan County**, 19 June 2020, Lang Li et al. *2020082*, *2020083* & *2020086* (HITBC); 20 June 2020, Lang Li et al. *2020091*, *2020092*, *2020093*, *2020094*, *2020095 & 2020096* (HITBC); 24 August 2020, Lang Li and Guan-long Cao, *2020127* & *2020128* (HITBC); 2 August 2022, Lang Li et al. *2022034*, *2022035* & *2022036* (HITBC).

## Supplementary Material

XML Treatment for Alseodaphnopsis hokouensis

## ﻿Additional information

### Author ORCIDs

Lang Li https://orcid.org/0000-0002-7439-2030

Jie Li https://orcid.org/0000-0001-8067-749X

### Data availability

All of the data that support the findings of this study are available in the main text.
